# Plasma Cathepsin D Activity Rather Than Levels Correlates With Metabolic Parameters of Type 2 Diabetes in Male Individuals

**DOI:** 10.3389/fendo.2020.575070

**Published:** 2020-09-30

**Authors:** Lingling Ding, Tom Houben, Yvonne Oligschlaeger, Albert V. Bitorina, Bart J. Verwer, Maarten E. Tushuizen, Ronit Shiri-Sverdlov

**Affiliations:** ^1^ Department of Molecular Genetics, School of Nutrition and Translational Research in Metabolism (NUTRIM), Maastricht Universtiy, Maastricht, Netherlands; ^2^ Department of Gastroenterology and Hepatology, Leiden University Medical Center, Leiden, Netherlands

**Keywords:** insulin resistance, metabolism, plasma cathepsin D activity, plasma levels of cathepsin D, plasma pH, type 2 diabetes

## Abstract

**Objective:**

Type 2 diabetes mellitus is a metabolic disorder characterized by insulin resistance. Previous studies in patients demonstrated that plasma levels of cathepsin D (CTSD), which is optimally active in the acidic environment of lysosomes, correlate with insulin resistance. As plasma pH is slightly reduced in type 2 diabetic patients and we have previously shown that plasma CTSD activity is causally linked to insulin levels *in vivo*, it is likely that the activity of CTSD in plasma will be increased in type 2 diabetes compared to healthy individuals. However, so far the interaction between CTSD activity and levels to postprandial metabolic derangements in type 2 diabetes is not known.

**Methods:**

Eighteen type 2 diabetes and 16 age-matched healthy males were given 2 consecutive standardized mixed meals, after which blood samples were collected. Plasma metabolic parameters as well as CTSD levels and activity were measured, and changes in plasma pH was assessed.

**Results:**

In line with the elevation of plasma free fatty acids (FFA) levels in male type 2 diabetics patients, plasma pH in type 2 diabetic individuals was decreased compared to male healthy individuals. While plasma CTSD levels were similar, plasma CTSD activity was increased in male type 2 diabetic compared to male healthy individuals. Besides, plasma CTSD activity rather than levels significantly correlated with indicators of type 2 diabetes (HbA1c, HOMA-IR and glucose). Furthermore, FFA was also independently associated with plasma CTSD activity (standardized β = 0.493, p = 0.007).

**Conclusions:**

Despite similar plasma CTSD levels, type 2 diabetic male individuals showed increased plasma CTSD activity compared to healthy males, which was independently linked to plasma FFA levels. Our data therefore point toward plasma CTSD as a metabolic regulator in male type 2 diabetes.

## Introduction

Diabetes mellitus is a global public health concern currently affecting more than 425 million people worldwide (1). Type 2 diabetes mellitus, accounting for approximately 90% of all diabetes mellitus cases, is hallmarked by insulin resistance, a pathological phenomenon where cells fail to respond to insulin to increase glucose uptake and utilization (2, 3). However, though certain metabolic cascades have been unraveled, insight into the complete metabolic picture of insulin resistance is still lacking as it is unclear why certain patients do and others do not develop insulin resistance. In order to identify those patients at increased risk for type 2 diabetes development, it is of utmost importance to improve our understanding of the metabolic mechanisms involved with insulin resistance.

A key mechanism involved with the development of insulin resistance is lipotoxicity, an umbrella term used to identify the deleterious effects of access lipid storage in non-adipose tissue (4). Lysosomes are essential cell organelles mediating lipid degradation, implying that under lipotoxic conditions (for example during increased cellular lipid influx) lysosomal function is compromised. Indeed, lipid-mediated damage to lysosomal membranes resulted in the unintended translocation of lysosomal enzymes into the cytosol and/or the extracellular environment (5–9). In line with these observations, we and others previously showed that accumulation of specifically oxidized lipids in lysosomes leads to the extracellular secretion of aspartic lysosomal enzyme cathepsin D (CTSD) (10, 11). Likewise, plasma CTSD levels have been previously correlated with insulin resistance in newly diagnosed type 2 diabetes (12) and two large community cohorts without diabetes (with more than 70% of participants being healthy residents) (13) and were even implicated as non-invasive markers for different stages of NAFLD, a metabolic condition linked to type 2 diabetes (10, 14). Relevantly, the enzymatic activity of CTSD is optimally active in an acidic environment, which is maintained *via* pH regulation (15). pH reductions in the interstitial fluid of diabetic patients (16) therefore imply changes in plasma CTSD activity in type 2 diabetes, which might result in metabolic changes. Furthermore, while insulin-treated diabetic rats showed increased CTSD activity in liver, kidney, heart and brain (17), inhibition of plasma CTSD activity in a steatotic rat model reduced plasma insulin levels (18). Thus, these evidences provide support for a potential functional metabolic link between insulin resistance and plasma CTSD activity.

To validate this view, in the current study we investigated whether type 2 diabetic patients have increased plasma CTSD activity compared to healthy individuals and whether plasma CTSD levels and activity link to postprandial metabolic parameters of type 2 diabetes. For this aim, plasma pH, plasma CTSD levels and activity as well as type 2 diabetes-related plasma parameters were measured in eighteen male type 2 diabetic patients and sixteen age-matched healthy males in the postprandial state. Our study demonstrated that plasma pH decreased in male type 2 diabetic individuals as compared to male healthy individuals, which can be partly explained by increased plasma FFA levels in type 2 diabetic patients. Additionally, compared to male healthy individuals, type 2 diabetic males demonstrated increased plasma CTSD activity. Furthermore, we observed that plasma CTSD activity, and not levels, significantly correlated with indicators of type 2 diabetes (plasma HbA1c, HOMA-IR and glucose) in males. Moreover, plasma CTSD activity also independently associated with a metabolic parameter of type 2 diabetes (plasma free fatty acids (FFA) in males). Altogether, our observations show that, despite similar plasma CTSD levels, type 2 diabetic male individuals showed increased plasma CTSD activity compared to healthy males, which was independently linked to plasma FFA levels. As FFA reduced the pH and pH was reduced in type 2 diabetic males, pH potentially played a central role in the activity of CTSD. Our data therefore point for the first time toward plasma CTSD as a metabolic regulator in male type 2 diabetes.

## Materials and Methods

### Subject Characteristics

The study populations comprised of 34 Caucasian males, aged between 40 and 65 years, which were classified as age-matched healthy controls (n = 16) and type 2 diabetes (n = 18). All subjects were recruited by advertisement and gave written informed consent. Before and during recruitment, type 2 diabetic patients were only allowed to follow a diet or to take the glucose-lowering agents sulphonylurea and/or metformin. Exclusion criteria were excess alcohol intake (>20 units/wk), history of hepatitis and/or pancreatitis, abnormal liver and renal function tests (>2 times upper limits of normal), recent (<3 months) changes in weight (≥5%) and/or medication, history or current use of glucocorticosteroids, insulin and/or thiazolidinediones, statins or other lipid-lowering drugs. Twenty-four hours prior to examination, the subjects were refrained from heavy physical activities, and during the examination, subjects were instructed to omit their medication. The present study was approved by the ethical committee of VU University and the study protocol was in accordance with the ethical guidelines of the declaration of Helsinki (1975).

### Study Design

After an overnight fast, all subjects consumed two consecutive standardized mixed meals as breakfast and lunch (4 h later), within 15 min. The meals were isocaloric (900 kcal), containing 75 g carbohydrates, 50 g fat (60% saturated) and 35 g protein. Subsequently, blood samples were collected from the antecubital vein 2 h following lunch as previously described (19).

### Biochemical Measurements of Plasma Parameters

Plasma glucose concentrations were measured by a hexokinase-based technique (Roche Diagnostics, Mannheim, Germany) and insulin concentrations by an immunoradiometric assay (Centaur, Bayer Diagnostics, Mijdrecht, The Netherlands). Plasma total cholesterol, high-density lipoproteins (HDL), triglycerides (TG), free fatty acids (FFA), gamma glutamyl transferase (GGT) and alanine transaminase (ALT) were determined by enzymatic methods (Modular, Hitachi, Japan). Low-density lipoproteins (LDL) were calculated by the Friedewald formula. Glycated hemoglobin (HbA1c) was measured by means of cation exchange chromatography (Menarini Diagnostics, Florence, Italy; reference values: 4.3–6.1%). Plasma remnants-like particle cholesterol (RLP cholesterol) was analyzed using an immuno-separation assay (Otsuka Pharmaceutical Co., Tokyo, Japan). The homeostasis model assessment of insulin resistance (HOMA-IR) was calculated as fasting insulin (μU/ml) × glucose (mmol/l)/22.5 as described by Matthews et al. (20). Plasma apoB-48 and apoB-100 were measured using a sandwich ELISA method as previously described (21). Plasma lactate concentration was measured *via* the lactate assay kit according to the protocol (Sigma-Aldrich, Netherlands). Plasma CTSD levels were determined by an enzyme-linked immunosorbent assay (USCN Life Science, Wuhan, China) and plasma CTSD activity was measured using a CTSD activity assay kit (MBL International, Woburn, USA), according to the manufacturer’s protocols.

### Plasma pH Measurements

The impact of plasma pH on plasma CTSD activity was assessed by using pH-adjusted reaction buffer, i.e., pH 4.0 (mimicking lysosomal pH) and pH 7.0 (mimicking plasma pH), respectively. Further, the pH of pooled plasma derived from healthy individuals *versus* type 2 diabetes patients was determined using a Seahorse Bioscience XF96 Extracellular Flux Analyzer (Aligent, USA). While pH measurements should ideally have been performed on individual plasma samples, we performed the pH measurement on pooled plasma samples from healthy controls and type 2 diabetic patients due to the low available plasma volumes. Additionally, to investigate a change of CTSD activity within a relevant plasma pH change, we measured plasma CTSD activity after adjusting the pH from pH 7.4 to pH 7.1. Furthermore, the effect of FFA (600 μM palmitate; Sigma Aldrich, Netherlands) on plasma pH was assessed *in-vitro* using plasma derived from random healthy volunteers. The palmitate stock (1,800 μM, the ration of palmitic acid: bovine serum albumin (BSA) = 6:1; BSA from MP Biomedicals, Netherlands) was prepared in 1.25× MKR (Modified Krebs Ringer Buffer in MQ water) with adjusted pH of 7.5. To get 600 μM FFA, 100 μl FFA stock was added into 200 μl plasma. Additionally, due to the influence of coagulant-EDTA, storage time and processing of blood samples (i.e., centrifuge) on plasma pH (20, 21), the absolute pH value are higher than 7.4.

### Statistical Analyses

Statistical analyses were performed using SPSS 24.0 (IBM, Armonk, NY, USA) and Graphpad Prism 6.0 for Microsoft Windows. All data were expressed as means ± SEM. The differences in subjects’ characteristics were tested using independent sample t-test in SPSS. The differences between two groups for the plasma pH experiment were tested using unpaired *t-test* in Graphpad Prism 6.0. Spearman’s correlations determined simple correlations between plasma CTSD levels/activity and parameters related to type 2 diabetes. Subsequently, multiple linear regression analyses were performed, in which plasma CTSD levels/activity served as dependent variable in model 1 (simple regression), model 2 (model 1 + adjustment for age), model 3 (model 2 + adjustment for BMI) and model 4 (model 3 + adjustment for waist). *p*-value <0.05 was considered statistically significant.

## Results

### General Characteristics of Healthy Controls and Type 2 Diabetic Subjects

Thirty-four males were enrolled in the study, consisting of 16 healthy controls and 18 type 2 diabetes with a mean age of 56.8 and 55.1 years, respectively. General characteristics (**Table 1**) show that BMI, waist, systolic (SBP) and diastolic blood pressure (DBP) were significantly higher in male patients with type 2 diabetes compared to healthy males. Plasma levels of glucose, HbA1c and insulin as well as HOMA-IR were significantly higher in male type 2 diabetic patients compared to male healthy controls, pointing toward disturbed glucose metabolism in male type 2 diabetic patients. Plasma lactate in type 2 diabetic male patients was also significantly higher than healthy males. No significant differences were found in postprandial plasma TG, RLP-cholesterol, ApoB48 and ApoB100 between the male healthy controls and type 2 diabetes. Plasma FFA levels significantly increased, while plasma HDL significantly decreased in type 2 diabetes compared to healthy individuals, indicating disturbed lipid metabolism in male type 2 diabetic patients. In line, plasma levels of ALT, GGT and hs-CRP were significantly higher in male type 2 diabetic patients than healthy males.

**Table 1:** General characteristics of healthy controls and type 2 diabetic subjects.

	Parameters	Control	T2DM	*p*
**Basic factors**	Number, N	16	18	
	Age (year)	56.8 ± 1.9	55.1 ± 1.3	0.477
	BMI (kg/m^2^)	26.9 ± 0.7	32.9 ± 1.1	0.000*
	Waist (cm)	98.3 ± 2.2	113.9 ± 2.8	0.000*
	SBP (mmHg)	122.9 ± 2.1	136.0 ± 3.3	0.003*
	DBP (mmHg)	76.3 ± 1.7	84.1 ± 1.2	0.001*
**Glucose-related parameters**	Glucose (mmol/L)	6.01 ± 0.19	8.75 ± 0.51	0.000*
	HbA1c (%)	5.53 ± 0.08	6.74 ± 0.16	0.000*
**Insulin-related parameters**	Insulin (pmol/L)	147.04 ± 18.18	295.58 ± 33.46	0.001*
	HOMA-IR	0.99 ± 0.14	4.06 ± 0.56	0.000*
	Lactate (mmol/L)	0.51 ± 0.03	0.70 ± 0.05	0.008*
**Lipid-related parameters**	TG (mmol/L)	2.36 ± 0.33	3.01 ± 0.40	0.221
	FFA (mmol/L)	0.24 ± 0.01	0.32 ± 0.02	0.002*
	HDL (mmol/L)	1.26 ± 0.08	0.90 ± 0.04	0.007*
	RLP-Chol (mg/ml)	13.21 ± 1.56	13.65 ± 1.72	0.850
	ApoB100 (mg/dl)	110.29 ± 6.89	124.87 ± 6.01	0.121
	ApoB48 (µg/ml)	15.38 ± 1.78	13.87 ± 1.27	0.493
**Liver indicators**	ALT (U/L)	20.00 ± 2.25	32.29 ± 3.55	0.007*
	GGT (U/L)	21.31 ± 2.78	33.00 ± 3.08	0.010*
**Inflammation**	Hs-CRP (mg/L)	0.85 ± 0.16	1.92 ± 0.31	0.006*

Data are mean ± SEM.

T2DM, type 2 diabetes mellitus; BMI, body mass index; SBP, systolic blood pressure; DBP, diastolic blood pressure; HbA1c, hemoglobin; HOMA-IR, homeostasis model assessment of insulin resistance; TG, triglyceride; FFA, free fat acids; HDL, high-density lipid protein; RLP-chol, remnants like particle cholesterol; GGT, gamma-glutamyl transpeptidase; ALT, alanine transaminase; Hs-CRP, high sensitive C-reaction protein; p: Control vs T2DM. *p < 0.05 is statistically significant.

### Reduced Plasma pH in Male Type 2 Diabetic Patients Due to Elevated FFA Levels

**Figure 1:**
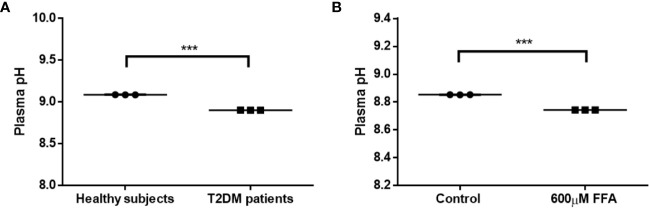
Reduced plasma pH in male type 2 diabetes due to elevated FFA levels. **(A)** Plasma pH in T2DM male individuals compared to male healthy subjects, **(B)** the effect of high concentrations of FFA (600 µM) on plasma pH. Data are mean ± SEM. ***p < 0.001. T2DM, type 2 diabetes mellitus.

To confirm whether type 2 diabetes have lower plasma pH compared with healthy individuals, we measured the pH of pooled plasma from male healthy individuals *versus* male type 2 diabetes. Our data showed a significant reduction in plasma pH of male type 2 diabetes compared to healthy males (**Figure 1A**; pH reduction = −0.19, p < 0.001), though the pH values were outside of the physiological range. Next, *ex-vivo* settings were tested using plasma derived from healthy volunteers to investigate whether the slight decline in plasma pH in type 2 diabetic patients is related to increased levels of FFA, which is known to play a key role in the pathology of type 2 diabetes (22). As expected, treatment with FFA (600 μM) resulted in a significant reduction in plasma pH (**Figure 1B**). Altogether, these data show the potential of FFA to significantly impact the plasma pH, which might influence plasma CTSD activity in type 2 diabetes.

### Plasma CTSD Activity Is Superior to Levels in Distinguishing Type 2 Diabetes From Healthy Controls in Male Individuals

**Figure 2:**
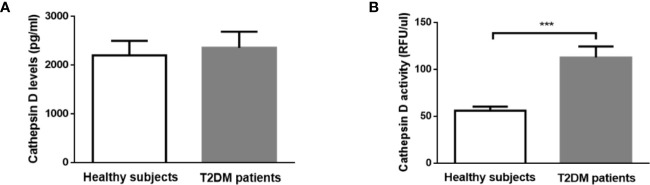
In contrast to CTSD level, CTSD activity distinguishes between healthy controls and T2DM in male individuals. **(A)** CTSD levels, **(B)** CTSD activity. Data are mean ± SEM. ***p < 0.001. T2DM, type 2 diabetes mellitus.

To confirm that plasma CTSD indeed maintains reduced activity outside of the lysosomes, the activity of plasma CTSD was measured at pH 7 (physiological pH) and at pH 4 (lysosomal pH). Our data demonstrated that plasma CTSD maintained ~50% of its proteolytic activity at neutral pH compared to acidic pH (**Figure S1**). Next, based on the above observations, plasma CTSD activity and levels were measured to compare the difference between type 2 diabetes and healthy subjects in males. No significant differences in plasma CTSD levels were observed between male type 2 diabetes and healthy individuals (**Figure 2A**). In line with the reduced plasma pH, plasma CTSD activity was significantly higher in male type 2 diabetic patients compared to male healthy individuals (**Figure 2B**, p<0.001), suggesting that plasma CTSD activity is linked to type 2 diabetes progression. Likewise, as shown in **Figure S2**, we observed that plasma CTSD activity at pH 7.1 increased compared to pH 7.4, supporting a change of CTSD activity within a relevant plasma pH change. Additionally, while lactate levels were significantly higher in type 2 diabetic males, these levels did not correlate with plasma CTSD activity, suggesting that lactate likely did not influence plasma CTSD activity (data not shown).

### Plasma CTSD Activity Rather Than Levels Significantly Correlates With type 2 Diabetes Indicators in Male Individuals

**Figure 3:**
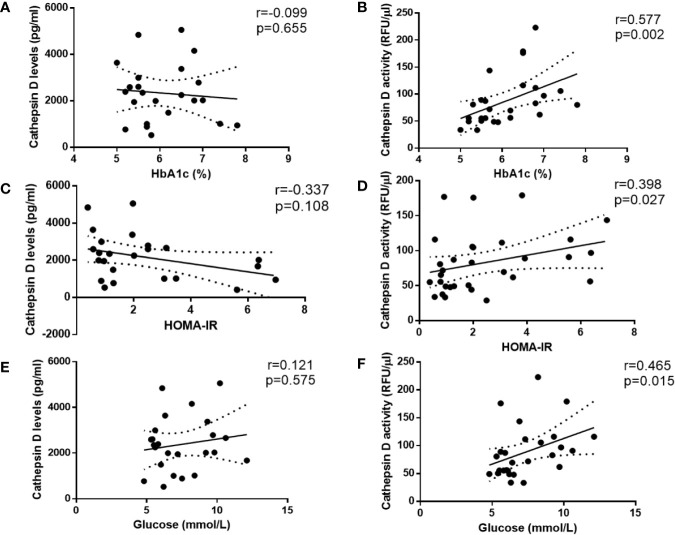
Spearman correlations between CTSD levels and activity versus type 2 diabetes-indicators in male individuals. CTSD levels **(A, C, E)** or activity **(B, D, F)** vs. HbA1c (%), HOMA-IR and glucose (mmol/L), respectively. Spearman’s correlations were performed. p < 0.05 is considered statistically significant.

To investigate whether plasma CTSD activity is correlated with type 2 diabetes indicators (i.e., HbA1c (%), HOMA-IR and glucose) compared with plasma CTSD levels, Spearman’s correlations were performed. In line with the finding that plasma CTSD activity was significantly higher in male type 2 diabetes compared with healthy males, we found that plasma CTSD activity positively correlated with HbA1c (%) (**Figure 3B**; r = 0.577, p = 0.002), HOMA-IR (**Figure 3D**; r = 0.398, p = 0.027) and plasma glucose (**Figure 3F**; r = 0.465, p = 0.015). In contrast, levels of plasma CTSD did not correlate with indicators of type 2 diabetes, including HbA1c (%), HOMA-IR and glucose (**Figures 3A, C, E**). Additionally, linear regression analyses were performed to evaluate whether the correlation between plasma CTSD activity and type 2 diabetic indicators (HbA1c (%), HOMA-IR and glucose) were dependent or independent of age, BMI and waist. As shown in **Table S1**, no significant associations were observed between plasma CTSD activity and HbA1c (%), HOMA-IR as well as glucose after adjustment of age, BMI and waist, indicating dependency of these parameters on the link between plasma CTSD activity and HbA1c (%)/HOMA-IR/glucose. Likewise, HbA1c (%), HOMA-IR and glucose were also not independently associated with plasma CTSD levels (data not shown). Taken together, our data indicate that plasma CTSD activity rather than levels correlates with type 2 diabetic indicators (plasma HbA1c, HOMA-IR and glucose), but their associations are dependent of age, BMI and waist.

### Metabolic Parameter Related to Type 2 Diabetes (FFA) Is Independently Associated With Plasma CTSD Activity in Male Individuals

**Figure 4:**
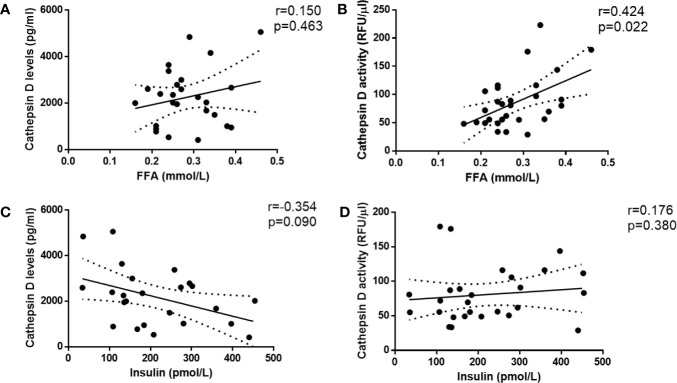
Plasma CTSD activity rather than levels significantly correlates with metabolic parameter of type 2 diabetes (FFA) in male individuals. CTSD levels **(A, C)** or activity **(B, D)** *vs.* FFA and insulin, respectively. Spearman’s correlations were performed. *p* < 0.05 is considered statistically significant.

Insulin and FFA play a central role in metabolic disturbances related to insulin resistance and type 2 diabetes. For this reason, we investigated whether plasma FFA and insulin are associated with plasma CTSD levels and activity. Firstly, Spearman’s correlation was performed to analyze the simple correlation between plasma CTSD levels/activity and metabolic parameters of type 2 diabetes (plasma FFA and insulin). As shown in **Figures 4A, B**, plasma CTSD activity, but not levels, correlated with plasma FFA (r = 0.424, p = 0.022). Both plasma CTSD levels and activity were not correlated with insulin (**Figures 4C, D**). To further evaluate whether these correlations were dependent or independent of age, BMI and waist, linear regression analyses were performed. As displayed in **Table 2**, plasma FFA positively associated with CTSD activity (standardized β = 0.494, p = 0.006), even after adjustment for age (Model 2: standardized ß = 0.492, p = 0.008), BMI (Model 3: standardized ß = 0.471, p = 0.008) and further correction for waist (Model 4: standardized ß = 0.493, p = 0.007). However, plasma insulin levels were not positively associated with CTSD activity even adjustment of age, BMI and waist (as shown in **Table S1**). Altogether, these data demonstrate that the metabolic parameter of type 2 diabetes (plasma FFA) is independently associated with plasma CTSD activity, linking plasma CTSD activity to insulin resistance *via* changes in FFA metabolism.

**Table 2:** Plasma FFA is independently (positively) associated with plasma CTSD activity in male individuals.

Dependent variable: CTSD activity			
Independent Variables	*p* value	FFA	R^2^
Models		Standardized coefficients β
**Model 1**	0.006	0.494	0.244
**Model 2**	0.008	0.492	0.250
**Model 3**	0.008	0.471	0.329
**Model 4**	0.007	0.493	0.349

Data were analyzed by linear regression models: Model 1, simple regression; Model 2, model 1 + adjustment for age; Model 3, model 2 + adjustment for BMI; Model 4, model 3 + adjustment for waist. p < 0.05 is statistically significant.

## Discussion

In the current study, we show that a metabolically-induced reduction of plasma pH in male type 2 diabetes patients correlates with increased plasma CTSD activity, which on its turn is linked to elevated plasma lipid and glucose levels. Our data therefore point toward plasma CTSD as a novel metabolic regulator in type 2 diabetes. These findings are in line with our previous studies that demonstrated a role for plasma CTSD activity in lipid metabolism and insulin resistance (18, 23, 24).

Under healthy conditions, CTSD has been demonstrated to play important roles in maintaining numerous physiological functions, including degradation of intracellular proteins, activation of hormones, growth factors and enzymatic precursors, hydrolysis of LDL cholesterol, and regulating programmed cell death (15). Besides physiology, CTSD has also been reported to be involved with the pathogenesis of a whole array of disorders such as in cancer, Alzheimer and metabolic syndrome related diseases (14, 25, 26). Indeed, emerging studies currently have focused on the role of CTSD in metabolic syndrome, linking CTSD to NAFLD (14) and type 2 diabetes (24). More specifically, increasing reports link CTSD activity to disturbances in lipid metabolism (18, 23), a finding which we could also confirm in here in our study. Therefore, with lipotoxicity as one of the key drivers for type 2 diabetes, CTSD might be an auspicious new player in the complex network contributing to insulin resistance by mechanisms that need to be further explored.

Our finding that plasma pH is influenced by changes in plasma FFA suggests an essential role for pH in regulating physiological processes related to metabolism. Indeed, enzyme activity is known to be highly dependent on the pH, a phenomenon that has been best described within the intralysosomal environment (27). Building further on this modulatory role of pH on enzyme activity, in this study, our findings imply that pH also influences enzyme activity in the plasma, suggesting the impact of pH also beyond the intralysosomal environment. However, as our pH measurements were outside of the physiological range, the link between FFA, pH and enzyme activity should be considered with caution at this stage as more research is necessary to confirm this claim. Nevertheless, a reduction of 0.05 units from the normal blood pH (being 7.40) in diabetic patients indeed results in acidosis (28), a condition which accelerates the progression of pathological features of diabetes by hampering the activity of metabolic enzymes such as phosphofructokinase (29). Furthermore, subtle reductions in pH also increase cathepsin activity in-vivo or ex-vivo in the context of cancer (30–33). Taken this modulatory function of pH on enzyme activity into account, it is possible that the plasma pH reduction in diabetic patients (16, 34, 35) also influenced CTSD activity in our study. Regardless, plasma pH has an essential modulatory function on metabolic processes by influencing enzyme activity and for this reason future studies should be done to clarify this role of pH on metabolic processes in more detail.

The observation that metabolic parameter (plasma FFA) related to type 2 diabetes associated with plasma CTSD activity (as dependent variable) in males implies an influence of this metabolic parameter on plasma CTSD activity. Indeed, considering pH as a key indicator for maintenance of enzyme activity, we here describe that plasma FFA changes plasma pH likely *via* the acidic characteristics associated with FFA (36), thereby influencing plasma CTSD activity. These findings raise the question whether this pH-mediated metabolic influence on plasma CTSD activity has functional consequences. Our findings that (1) type 2 diabetic patients have higher plasma CTSD activity compared to healthy controls and (2) plasma CTSD activity positively correlates with indicators of type 2 diabetes (HbA1c, HOMA-IR and glucose) point toward the link between plasma CTSD activity and disturbed glucose metabolism (or insulin resistance) in type 2 diabetes. Moreover, we recently found that inhibition of extracellular CTSD activity reduced plasma insulin levels and hepatic lipids in rats with hepatic steatosis (18). Likewise, our group also previously observed that inhibiting CTSD activity *via* pepstatin A (an aspartic lysosomal enzymes inhibitor) reduces the gene expression of CD36 (a transporter of FFA) (23) that also mediates the suppression of FFA on insulin signaling (37). This data thereby suggests that CTSD activity is likely involved with insulin signaling *via* regulating FFA metabolic pathways (i.e., CD36 transporter). Furthermore, previous reports have proven the ability of CTSD to proteolysis and influence the bioavailability of insulin-like growth factors (IGFs), factors that have been extensively linked to insulin resistance (38, 39). These observations place plasma CTSD activity at the center of metabolic programming as in this way, CTSD receives “signals” from metabolic factors such as FFA on one hand and regulates glucose metabolism *via* insulin or IGFs on the other hand. Therefore, CTSD should be further investigated as a central metabolic regulator in the context of type 2 diabetes and potentially other metabolic diseases.

While in contrast to a previous study (12), we did not observe changes in plasma CTSD levels in type 2 diabetes patients, we observed that type 2 diabetes showed increased plasma CTSD activity, which also correlated metabolic parameters of type 2 diabetes. As starvation influences the secretion of lysosomal enzymes, one potential explanation for the lack of correlation with CTSD levels might be related to the fact that subjects in our study did not undergo overnight fasting prior to blood sampling (40, 41), and as such the differences in plasma CTSD levels were below detection levels. Another explanation for the lack of correlation between plasma CTSD levels and type 2 diabetes in the current study could be related to the known effect of estrogen in increasing CTSD expression (42) as the previous report included both males and females (12), whereas all the subjects in our study are males. It is also noteworthy to mention that in our cohort, it cannot be completely excluded that the increase in CTSD activity is due to the presence of other diseases that coincide with type 2 diabetes (such as NAFLD). As plasma ALT levels, however, did not correlate with plasma CTSD activity (data not shown), it seems unlikely that the presence of NAFLD influenced our results.

In the current study, the identification of CTSD as a metabolic regulator might have implications for improving type 2 diabetes treatment. While a variety of drugs have currently been developed to treat type 2 diabetes (43–45), they are often featured by high costs and/or serious sides effects (43). Considering its functions as a metabolic regulator, CTSD-related strategies to treat type 2 diabetes may provide a relevant alternative for these existing anti-diabetic agents. A previous study by our group demonstrated that inhibiting circulating CTSD activity reduces plasma insulin levels in steatotic rats (18), confirming the potential therapeutic value of directly targeting CTSD to improve insulin sensitivity. However, to fully disclose CTSD and related pathways as a new therapeutic alternative to treat type 2 diabetes, additional and larger cohorts are essential to verify these results, and more in depth investigation into the mechanisms of CTSD are necessary. Nevertheless, our observations implying the functional role of CTSD as a metabolic regulator holds clinical value as it can lead to new ways to treat type 2 diabetes.

## Data Availability Statement

The raw data supporting the conclusions of this article will be made available by the authors, without undue reservation.

## Ethics Statement

The studies involving human participants were reviewed and approved by: The ethical committee of VU University Medical Center. The patients/participants provided their written informed consent to participate in this study.

## Author Contributions

RS-S and MT were responsible for concept and design of study and final review of manuscript. LD, TH, and YO were responsible for drafting of manuscript, data collection, statistical analysis, and critical review of manuscript. AB and BV were responsible for helping data collection and analysis. All authors contributed to the article and approved the submitted version.

## Funding

This study is funded by the Dutch Organization for Scientific Research (NWO) (Vidi grant no. 016.126.327), ASPASIA (grant no. 015.008.043), TKI-LSH (grant no. 40-41200-98-9306) and VCK (grant no. Swu16.0057-VT), and by a grant from the Dutch Diabetes Foundation (Grant number 2000.00.025). LD is supported by the Chinese Scholarship Council with file number 201709110146.

## Conflict of Interest

The authors declare that the research was conducted in the absence of any commercial or financial relationships that could be construed as a potential conflict of interest.

## Abbreviations

ALT, alanine transaminase; CTSD, Cathepsin D; FFA, free fatty acids; GGT, gamma glutamyl transferase; HDL, high-density lipoproteins; LDL, lowdensity lipoproteins; TG, triglycerides.
