# Supplementary material for: Plasma Cathepsin D Activity Rather Than Levels Correlates With Metabolic Parameters of Type 2 Diabetes in Male Individuals
Source: Front Endocrinol (Lausanne). 2020 Sep 30;11:575070. doi: 10.3389/fendo.2020.575070 (PMC7554511; doi:10.3389/fendo.2020.575070)

Supplementary Information

**Supplementary Table S1. Plasma HbA1c (%), HOMA-IR, glucose and insulin are not independently associated with plasma CTSD activity.**

| **Dependent variable: CTSD activity** |  |  |  |  |  |  |  |  |  |  |  |  |
| --- | --- | --- | --- | --- | --- | --- | --- | --- | --- | --- | --- | --- |
| **Independent Variables** |  | **HbA1c (%)** |  |  | **HOMA-IR** |  |  | **Glucose** |  |  | **Insulin** |  |
| **Models** | ***p***  **value** | **Standardized coefficients β** | **R^2^** | ***p***  **value** | **Standardized coefficients β** | **R^2^** | ***p* value** | **Standardized coefficients β** | **R^2^** | ***p* value** | **Standardized coefficients β** | **R^2^** |
| **Model 1** | 0.017 | 0.463 | 0.214 | 0.221 | 0.268 | 0.049 | 0.050 | 0.381 | 0.145 | 0.552 | 0.120 | 0.014 |
| **Model 2** | 0.022 | 0.457 | 0.217 | 0.219 | 0.282 | 0.050 | 0.066 | 0.377 | 0.145 | 0.572 | 0.117 | 0.016 |
| **Model 3** | 0.176 | 0.337 | 0.239 | 0.962 | -0.014 | 0.098 | 0.234 | 0.251 | 0.231 | 0.548 | -0.161 | 0.112 |
| **Model 4** | 0.108 | 0.409 | 0.295 | 0.969 | -0.012 | 0.098 | 0.118 | 0.344 | 0.295 | 0.558 | -0.165 | 0.112 |

Data was analyzed by linear regression models: Model 1, simple regression; Model 2, model 1 + adjustment for age; Model 3, model 2 + adjustment for BMI; Model 4, model 3 + adjustment for waist. p<0.05 is statistically significant.

**Supplementary Figure. S1. Plasma CTSD maintains reduced enzymatic activity at neutral pH.** Data is mean ± SEM. **p<0.01.


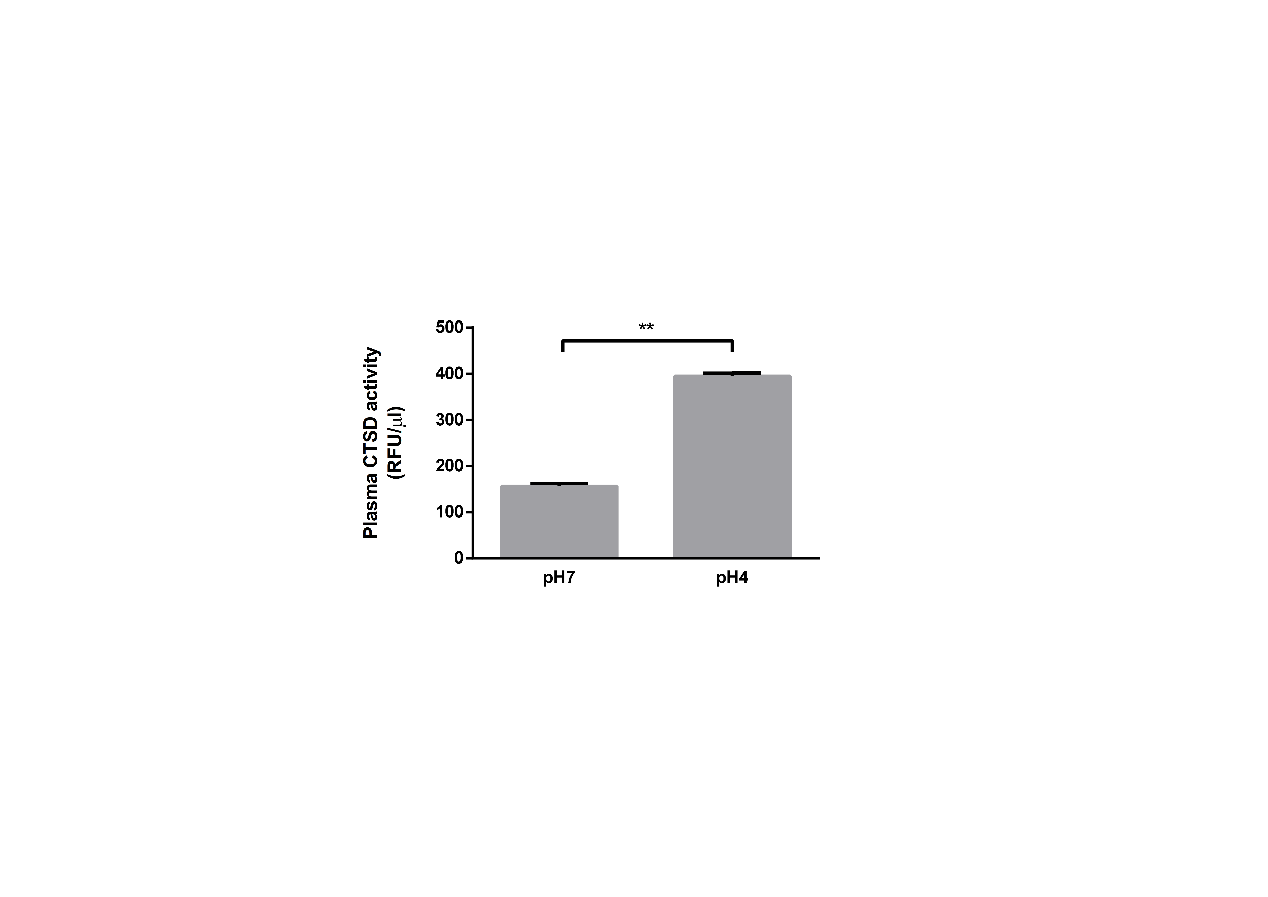


**Supplementary Figure. S2. A change of CTSD activity within a relevant plasma pH range.**


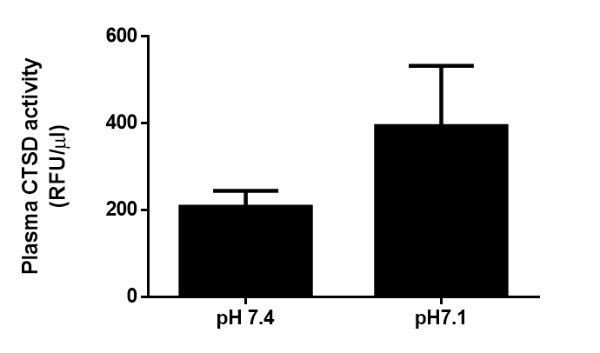

Supplement: Supplementary file 1 [file DataSheet_1.docx]
